# What drives junior doctors to use clinical practice guidelines? A national cross-sectional survey of foundation doctors in England & Wales

**DOI:** 10.1186/s12909-015-0510-3

**Published:** 2015-12-21

**Authors:** L. Manikam, A. Hoy, H. Fosker, Martin Ho Yin Wong, J. Banerjee, M. Lakhanpaul, A. Knight, P. Littlejohns

**Affiliations:** Population, Policy and Practice, UCL Institute of Child Health, 30 Guildford Street, London, WC1N 1EH UK; National Institute for Health and Care Excellence, 10 Spring Gardens, London, SW1A 2BU UK; University Hospitals of Leicester NHS Trust, Infirmary Square, Leicester, LE1 5WW UK; University College London Hospitals NHS Foundation Trust, 235 Euston Road, London, NW1 2BU UK; Department of Primary Care and Public Health Sciences, King’s College London, Addison House, London, SE1 IUL UK; King’s Improvement Science, Health Service and Population Research Department, Institute of Psychiatry, Psychology & Neuroscience, King’s College London, Addison House, London, SE1 IUL UK

**Keywords:** Clinical practice guidelines, Junior doctors, Evidence-based medicine

## Abstract

**Background:**

Clinical practice guidelines (CPGs) aim to improve patient care, but their use remains variable. We explored attitudes that influence CPG use amongst newly qualified doctors.

**Methods:**

A self-completed, anonymous questionnaire was sent to all Foundation Doctors in England and Wales between December 2012 and May 2013. We included questions designed to measure the 11 domains of the validated Theoretical Domains Framework (TDF). We correlated these responses to questions assessing current and future intention to use CPGs.

**Results:**

A total of 13,138 doctors were invited of which 1698 (13 %) responded. 1,035 (62.5 %) reported regular CPG use with 575 (34.4 %) applying CPGs 2–3 times per week. A significant minority of 606 (36.6 %) declared an inability to critically appraise evidence.

Despite efforts to design a questionnaire that captured the domains of the TDF, the domain scales created had low internal reliability. Using previously published studies and input from an expert statistical group, an alternative model was sought using exploratory factor analysis. Five alternative domains were identified. These were judged to represent: “confidence”, “familiarity”, “commitment and duty”, “time” and “perceived benefits”.

Using regression analyses, the first three were noted as consistent predictors of both current and future intentions to use CPGs in decreasing strength order.

**Conclusions:**

In this large survey of newly qualified doctors, “confidence”, “familiarity” and “commitment and duty” were identified as domains that influence use of CPGs in frontline practice. Additionally, a significant minority were not confident in critically appraising evidence.

Our findings suggest a number of approaches that may be taken to improve junior doctors’ commitment to CPGs through processes that increase their confidence and familiarity in using CPGs.

Despite limitations of a self-reported survey and potential non-response bias, these findings are from a large representative sample and a review of existing implementation strategies may be warranted based on these findings.

## Background

The aim of evidence-based clinical practice guidelines (CPGs) issued by the National Institute for Health and Care Excellence (NICE) is to improve and standardise quality of care delivered in the National Health Service (NHS) in England and Wales [1]. In the NHS, consultants and general practitioners (GPs) are responsible for leading the interpretation and implementation of NICE guidelines in clinical practice amongst doctors [2]. In line with the General Medical Council (GMC) publication “Tomorrow’s Doctors”, the current standard for United Kingdom (UK) medical education, there is an expectation that UK trained junior doctors both apply clinical practice guidelines and deliver evidence-based care [3].

However, despite the impetus, we do not know if there has been much change in the evidence indicating variable application of CPGs amongst senior clinicians [4]. A recent report from the GMC described factors that influenced clinicians acting in accordance with good practice [5]. A national survey of public health directors, from many years ago, noted the variable implementation of NICE CPGs with their full benefits remaining unrealised [6]. Finally, a comprehensive systematic review of responses by 11,611 clinicians, from 13 years ago, noted that more than a third of clinicians considered CPGs to be impractical, reduced physician autonomy and increased risk of litigation [7]. Anecdotally, these attitudes persist.

These opinions appear to be equally shared amongst future doctors with a national survey of medical students in England and Wales identifying views such as CPGs having negative influences on patient choice and decreasing practice autonomy. Furthermore, marked deficits in their knowledge of CPG development were identified [8]. In addition to the documented variation in the methods and content of evidence-based medicine (EBM) curriculum amongst UK medical schools, these perceptions are likely to be influenced by multiple factors such as supervising clinicians’ opinions, media reports and students' understanding of published material [9, 10].

As recent medical school graduates, it is possible that UK Foundation Doctors may share similar views. Recent research has identified national variation in the level of preparedness for clinical practice by Foundation Doctors [11]. It is therefore crucial to understand their use of CPGs and EBM in frontline clinical practice together with identifying barriers and enablers that may influence their implementation.

These tie in with a national report from the High Level Working Group on Evidence Based Clinical Effectiveness which emphasised the need for primary research incorporating behavioural theories across undergraduate students and practising physicians to understand the drivers that influence evidence - based practice (EBP) [2]. Building on our earlier national survey of medical students, we sought to investigate the extent of use of CPGs and EBM using the Theoretical Domains Framework (TDF).

The TDF is a validated framework to both assess healthcare professionals’ behaviours and inform interventions to change them [12]. It is commonly used within a four-step approach to designing a theory-informed implementation intervention. These steps consist of: (1) identifying the problem (who needs to do what differently), (2) assessing the problem (using the TDF to identify which barriers and enablers need to be addressed), (3) forming possible solutions (which intervention components could overcome the modifiable barriers and enhance the enablers) and (4) evaluating the selected intervention (how can behaviour change be measured and understood) [13, 14].

For example, to improve adherence to a CPG in acute low back pain management in primary care, the TDF was utilised to identify the barriers and enablers to the uptake of evidence into practice [15]. They were then categorised according to the behavioural domain that they operated. Intervention components (behaviour change techniques and modes of delivery) were then selected to overcome the modifiable barriers and enhance the enablers. These components were then combined into a cohesive intervention using the UK Medical Research Council (MRC) framework [15].

The TDF’s strengths lie in enabling a systematic approach starting from ascertaining target behaviours, identifying theoretical domains, matching behaviour change techniques, and finally designing an implementation intervention to address the behaviours [16]. However, it has been criticised for its subjectivity, its focus on behaviour rather than attitudes and the considerable time and resources required for intervention development [13]. Despite its censure, there is a rapidly increasing evidence base of its use in developing effective theory-informed implementation interventions [12–16].

Our study had three aims. First, we aimed to investigate the use of CPGs and EBM in frontline clinical practice amongst Foundation Doctors across different locations and specialties in England & Wales. Second, using the TDF, we sought to understand which behavioural domains correlated most strongly with current and future intentions to use CPGs [17]. Thirdly, we wanted to test the usefulness of the TDF through our study of self-reported behaviours.

## Methods

### Survey implementation

We conducted a self-reported, anonymous cross-sectional survey. Participants were Foundation Doctors based in the NHS in England and Wales (see box) working across a range of specialties (total population n = 13,138). The survey was undertaken between December 2012 and May 2013. A third party survey provider (SurveyMonkey) was used to disseminate the survey via a hyperlink to medical workforce deaneries and local education coordinators [18]. Participants were also given the option to complete a paper version of the questionnaire if preferred.

Three methods were utilised to maximise the response rate. First, all 14 regional Medical Workforce Deaneries in England & Wales were asked to forward an invitation e-mail to their doctors. As non-responders could not be identified, a reminder e-mail was sent to all Deaneries a month later to be cascaded. Secondly, every NHS hospital trust in England, and every Health Board in Wales was contacted (*n* = 167).

In each body, the postgraduate education programme coordinator was identified and approached to forward the survey invitation to Foundation Doctors employed by their local NHS hospital trust or Board. Thirdly, from the above two invitations, foundation doctors were invited and recruited (*n* = 64) to act as local survey promoters by disseminating questionnaires at local teaching sessions.

**Table:** 

**Foundation Doctors**
In the UK, all newly qualified doctors undertake the Foundation Programme, a mandatory two-year programme of general postgraduate medical training which forms the bridge between medical school and specialist/general practice training [19]. Foundation doctors rotate in six placements, each of four months in duration, through various specialties across a region, and through hospitals ranging from large university to small county hospitals. Over the programme, they are expected to build on their undergraduate medical education by gaining a breadth of experience on the use of EBM and CPGs in a variety of healthcare settings and to develop the skills necessary to appraise current evidence, in accordance with the UK GMC “Tomorrow’s Doctors” syllabus [3].

### Questionnaire development

The questionnaire which we validated and published in the national medical student survey was originally based on published aggregated survey responses to CPGs [6, 8]. As the questionnaire did not formally utilise the TDF, the questions asked could only be mapped to the TDF behavioural domains “Knowledge” and “Social/Professional Role and Identity” retrospectively. It was therefore necessary to expand the questionnaire to assess the 11 behavioural domains identified by the TDF (fully listed in Table 3 below) [12].

From our experience in developing the questionnaire utilised in the national medical student survey, five point Likert scales (responses were: 1 ‘Strongly disagree’, 2 ‘Disagree’, 3 ‘Unsure’, 4 ‘Agree’, and 5 ‘Strongly agree’) together with guidance on its use were deemed to be the most appropriate for inclusion in the questionnaire.

Questions from a previously published survey designed to assess current frequency and future intention to use CPGs utilising eight and four-point Likert scales were used respectively [20]. These were not converted to five point Likert scales due to its validation using those existing scales.

Questions were randomised with approximately half assessing positive views and the corresponding half assessing negative views towards EBM and CPGs. This was undertaken to minimise social desirability bias (i.e. tendency of survey respondents to answer questions in a manner viewed favourably by others) which have been reported in numerous other studies including a recent TDF - based study investigating midwives’ roles in smoking cessation during pregnancy [16].

The questionnaire also collected demographic information related to age, foundation year, ethnicity, gender, country, medical school of qualification, place of work, and current and future specialty of choice. Questionnaire development was undertaken in consultation with a health psychologist throughout the entire process.

### Questionnaire piloting

Several development iterations were undertaken during two rounds of piloting to produce a questionnaire that contained both a sufficient number of positive and negative items to minimise response bias and items to calculate a Cronbach’s alpha (measure of the internal reliability of a composite measure) for each domain. Respondent fatigue was assessed during each piloting round.

Piloting was undertaken by two groups of 19 Foundation Doctors (23 Foundation Year One, 6 Foundation Year Two and 9 unspecified) across 18 subspecialties from 7 Workforce Deaneries. Cronbach’s alpha was computed for each of the 11 domains.

Weak to moderate reliability scores were identified in the 1st pilot and thus a repeat pilot was subsequently undertaken utilising different questionnaire versions (i.e. positive and negative wording). Cronbach’s alpha values remained low.

Despite the low values, a decision was undertaken to maintain the same number of positively and negatively worded questions in the final survey from the 2nd pilot. There was the possibility that the relatively small number of students in the pilot samples had been unrepresentative with literature noting that the alpha scores might change when larger samples of participants are utilised [21]. There was also a desire for flexibility, so that the composition of scales could be adjusted if necessary to raise the alpha levels.

Interim versions consisted of a maximum of 53 questions with 5 questions per domain. The final version consisted of a total of 39 questions covering 11 domains with an average of approximately three questions per domain. The Supplement 1 file contains the final questionnaire used.

### Statistical analysis

Online responses, records and transcribed paper questionnaires were converted to International Business Machines (IBM) Statistical Package for Social Sciences (SPSS) Version 21.0 [22] for analysis. Items that had been negatively worded were reversed-coded prior to data analysis.

The responses are described through tabulation and proportions. Cronbach’s alpha scores were used to assess the internal reliability of the domains of the original TDF and those of the newly derived domains. A protocol diversion was undertaken when satisfactory Cronbach alpha values were not attained within the pre-specified TDF domains. In consultation with an expert statistical group and previously published studies, an exploratory (varimax) factor analysis was undertaken to derive alternative domains [23].

Multiple linear regression analysis was undertaken to explore which of the final domains correlated with self-reported behaviour on current frequency of CPG use and future use of CPGs. An analysis was also undertaken of the influence of demographic variables such as age, gender, time in practice, current specialty, intended future specialty and place of medical school graduation.

### Ethics

This was an anonymised, questionnaire study that was self-administered, with no feature that could identify the participants. Ethical review was undertaken and granted by the National Research Ethics Service (REF: 04/26/31).

## Results

### Characteristics of the respondents

The overall response rate was poor at 12.9 % (*n* = 1693). Of these respondents, 50.6 % (*n* = 855) were working as Foundation Year One doctors with most (93 %, *n* = 1509) graduating from a UK medical school. To assess the representativeness of the survey respondents to the national cohort of Foundation Doctors, a comparison between current specialties between respondents and national data (UK National Training Survey 2013) [24] was also undertaken using chi-squared tests (Table 1). These noted no significant differences amongst Foundation Year One (*X*^2^ = 7.390, df = 8, *p* = .495) and Two Doctors (*X*^2^ = 7.714, df = 11, *p* = .738).

**Table 1:** Specialty comparison between survey respondents and national data

	Year One		Year Two	
Category	Present survey results – *N* (%)	National training survey 2013 results (%)	Present survey results – *N* (%)	National training survey 2013 results (%)
Anaesthetics	20 (2.3)	2.6	14 (1.7)	3.1
Emergency medicine	22 (2.6)	2.1	92 (11.0)	16.8
General practice	0 (0.0)	0.0	170 (20.4)	15.9
Medicine	378 (44.3)	49.4	231 (27.7)	26.4
Obstetrics and gynaecology	18 (2.1)	1.3	43 (5.2)	4.9
Ophthalmology	4 (0.5)	0.1	3 (0.4)	0.8
Paediatrics and child health	27 (3.2)	2.6	35 (4.2)	5.6
Pathology	0 (0.0)	0.0	2 (0.2)	0.8
Psychiatry	35 (4.1)	1.8	32 (3.8)	4.6
Public health	0 (0.0)	0.0	13 (1.6)	0.6
Radiology	4 (0.5)	0.5	3 (0.4)	1.0
Surgery	270 (31.7)	39.5	135 (16.2)	19.5

### Domain creation prior to analysis

Our pre-specified analyses assumed that influences of CPG use amongst respondents would correlate with the TDF behavioural domains. Relatively low Cronbach’s alphas (Table 2) however were obtained for many of the domains (0.195 to 0.713). The TDF did not appear to be a good fit for our survey respondents.

**Table 2:** Factor loadings from exploratory factor analysis (varimax rotation). Values in bold correspond to the most significant results which were used to group the new domains.

Question	Question direction	Factor loadings									
		1	2	3	4	5	6	7	8	9	10
Q33 As a clinician, I have a duty to practise evidence-based medicine	Agreement with	**0.75**							0.184	0.145	
Q28 As a future senior clinician, I will need to serve as a role model in the practice of evidence - based medicine	Agreement with	**0.75**							0.12		
Q38 I intend to practise evidence - based medicine as it is best for the patient	Agreement with	**0.743**								0.153	
Q13 I am committed to practising evidence based medicine	Agreement with	**0.615**				0.201			0.132	0.233	
Q27 I want to use NICE guidelines to inform my every day clinical practice	Agreement with	**0.591**		0.176		0.23		0.143			
Q06 I intend to incorporate relevant NICE guidelines into my clinical work	Agreement with	**0.46**		0.156	0.106	0.447	0.118				0.126
Q24 I would feel guilty if I did not follow a NICE guideline and my patient's care was compromised as a result	Agreement with	**0.409**		0.186	0.16	0.222					
Q20 I find that evidence - based medicine is difficult to practise	Disagreement with		**0.305**	0.217	0.2		0.162	0.177	0.383	0.263	
Q35 I am confident I can implement NICE guidelines in clinical situations, even when the time available is very limited.	Agreement with		**0.602**	0.108	0.264	0.113	0.109			0.257	
Q23 Time pressures do not prevent me from keeping up to date with current guidelines	Agreement with		**0.757**								
Q29 Using NICE guidelines increases the time needed to manage patients	Disagreement with		**0.468**	0.423		0.114		0.141	0.221		
Q37 Time pressures prevent me from keeping up to date with current guidelines	Disagreement with		**0.742**				0.193				
Q21 Following NICE guidelines can improve patient clinical outcomes	Agreement with	0.511		**0.271**	0.175		0.107	0.12			
Q36 Senior clinicians' opinions have influenced me to be negative towards evidence - based medicine	Disagreement with	0.381		**0.204**			0.177		0.537		
Q08 As a clinician, using NICE guidelines increases the risk of litigation	Disagreement with	0.283		**0.534**			0.128		0.145		0.15
Q25 Implementing NICE guidelines can reduce healthcare costs	Agreement with	0.244		**0.525**	0.183						0.124
Q26 The overall effect of using NICE guidelines is to increase the complexity of clinical practice	Disagreement with	0.235		**0.628**					0.114		
Q39 Senior clinicians that I work with prioritise new guidelines over previous, older pathways	Agreement with	0.171			**0.621**			0.169	0.225		
Q03 NICE guidelines and recommendations prioritise clinical effectiveness over cost effectiveness	Agreement with	0.145			**0.202**	0.141		0.512			0.124
Q30 I am able to find relevant NICE guidelines to apply to my clinical work	Agreement with		0.191	0.175	**0.519**	0.197					0.11
Q32 I have a clear plan of action for implementing relevant NICE guidelines in clinical situations	Agreement with		0.369		**0.506**	0.311	0.147			0.114	
Q19 I am familiar with the content of the NICE guidelines relevant to my clinical area	Agreement with		0.258		**0.414**	0.362	0.153			0.35	
Q10 I find it easy to apply NICE guidelines to the complex circumstances of individual patients	Agreement with	0.15	0.357		0.195		**0.359**	0.214			
Q15 I am not expert enough to use NICE guidelines to influence clinical practice	Disagreement with			0.309		0.168	**0.435**	0.178	0.136	0.331	
Q11 I am scared of colleagues' reactions if I follow guidelines instead of senior advice	Disagreement with		0.201				**0.715**		0.102		
Q16 The complex circumstances of individual patients mean that NICE guidelines cannot be applied to many patients	Disagreement with			0.374	0.179		**0.259**	0.35		0.299	0.109
Q04 I refer more to handbooks than to guidelines for supporting my clinical practice	Disagreement with		0.208	0.177	0.146	0.206	**0.403**				0.237
Q09 My clinical decisions are influenced by senior advice rather than guidelines	Disagreement with		0.211			0.342	**0.532**	0.113			0.104
Q01 NICE guidelines and recommendations are developed in collaboration with clinicians	Agreement with	0.409		0.226	0.183			0.163			0.433
Q34 I can critically appraise evidence relevant to my clinical practice	Agreement with	0.252	0.147							0.751	
Q18 The senior clinicians who I work with create a learning environment for juniors where evidence-based practice is discouraged	Disagreement with	0.21		0.156	0.157				0.663		
Q12 I reflect on my clinical practice and try to identify occasions when I could have better followed NICE guidelines	Agreement with	0.16				0.694					
Q02 I am able to maintain a good doctor-patient relationship even when following NICE guidelines means denying a patient a treatment they want	Agreement with	0.149			0.149			0.558			
Q07 NICE guidelines and recommendations do not have direct input from the lay public	Disagreement with	0.147									0.759
Q05 I try to encourage my peers to follow NICE guidelines where applicable	Agreement with	0.127			0.212	0.701	0.138				
Q22 The overall effect of using NICE guidelines is to decrease the complexity of clinical practice	Agreement with	0.114		0.178	0.276		0.176	0.133			
Q31 Senior clinicians seem to want care to be influenced more by NICE guidelines than guidelines published by other groups (i.e. SIGN, European Respiratory Society)	Agreement with		0.163		0.451						
Q14 Using NICE guidelines can decrease patient choice	Disagreement with			0.16	−0.141		0.107	0.691			
Q17 NICE guidelines and recommendations have stakeholder input from drug companies	Agreement with			−0.533	0.148		0.133	−0.207			

(Factor loadings depicted = > .10)

In consultation with the King's College London (KCL) Department of Primary Care and Public Health Sciences Statistical Group, an exploratory factor analysis (varimax rotation) was undertaken to identify response patterns which could be categorised into alternative domains. Table 2 below contains details of factor loadings.

These factor loadings were considered by the research team in conjunction with scree plots. The derived factors were not the result of pre-specified cut-off loadings, but instead were selected on the basis of easily interpretable domains with satisfactory Cronbach alpha values. Five domains were identified and labelled as: “confidence”, “familiarity”, “commitment and duty”, “time” and “perceived benefits”.

These Cronbach’s alpha values (Table 3) ranged from 0.58 (confidence) to 0.79 (commitment and duty). Not all domain scales could be created using a mixture of positive and negative items with the first and third domains consisting completely of positive items. In addition, the fourth domain was composed of five negative items and only one positive item. Factor analysis with an oblique rotation was also undertaken however this did not identify any interpretable response patterns.

**Table 3:** Comparison between TDF and factor analysis-derived domains

Theoretical Domains Framework (TDF)	
Domain label	Cronbach’s alpha from this sample
Knowledge	.316
Skills	.295
Social/professional role and identity	.713
Beliefs about capabilities	.430
Beliefs about consequences	.481
Motivation and goals	.698
Memory, attention, and decision processes	.423
Environmental context and resources	.578
Social influences	.350
Emotion	.185
Behavioural regulation	.559
Domains derived through factor analysis	
Domain label	Cronbach’s alpha from this sample
Confidence	.578
Familiarity	.595
Commitment and duty	.787
Time	.677
Perceived benefits	.598

In the varimax rotation-derived results, questions which formed parts of the newly derived domains were then aggregated through simple addition, producing domain scores that ranged from 5 to 35 in the case of the largest (seven item) domain. Out of the original list of 39 potential “domain” questions in the questionnaire, 11 were not selected for use in any scale.

### Use of CPGs and EBM in frontline clinical practice amongst Foundation Doctors

Majority (62.5 ± 2.2 %, 1035 out of 1656) of respondents reported consistent use of CPGs for most patients. The commonest frequency of CPG use was reported as 2–3 times per week for 34.4 ± 2.1 % (575 out of 1672). Notably, a minority utilised CPGs less than once per week (18.6 ± 1.7 %, 311 out of 1672). A total of 63.4 ± 2.1 % (1050 out of 1656) of doctors were able to critically appraise evidence relevant to their clinical practice (i.e. Strongly agreed or agreed with the relevant question).

The majority 76.4 ± 1.9 % (1280 out of 1676), of doctors were influenced by senior advice with 49.6 ± 2.2 % (831 out of 1675) of doctors influenced by handbooks rather than CPGs. 86.0 ± 1.5 % (1437 out of 1670) of doctors reported that using CPGs reduced the risk of litigation whilst 29.4 ± 2.0 % (489 out of 1664) of doctors noted that CPG use reduced patient choice. A minority (22.9 ± 1.9 %, 382 out of 1670) of doctors believed that CPGs are inapplicable due to the complex circumstances of patients with 25.5 ± 1.9 % (425 out of 1668) of doctors noting that CPGs increased clinical practice complexity.

A proportion of doctors (43.9 ± 2.2 %, 736 out of 1674) reported being scared of colleagues’ reactions if they followed CPGs instead of senior advice; 76.6 ± 1.9 % (1277 out of 1668) of doctors stated they would feel guilty if a patient’s care was compromised as a result of CPG non-adherence. Whilst a majority of doctors (86.6 ± 1.5 %, 1448 out of 1673) were committed to EBP, a minority of doctors (18.7 ± 1.7 %, 313 out of 1670) found it difficult to practise. An even smaller minority (7.8 ± 1.5 %, 1340 out of 1675) stated that senior clinicians discouraged EBP.

Subgroup analyses by demographic variables were undertaken to ascertain the presence of any variation in the domain scores. T-tests and correlation coefficients were utilised. No difference by gender, time in practice (i.e. Foundation Year One or Two), current specialty, intended future specialty or place of medical training were identified. A small but statistically significant association between “confidence” and age (*r* = .115, *p *<. 0001, *n* = 1582) was noted. Due to the number of subgroup analyses undertaken, this is likely due to a Type 1 error.

### Domains that correlated most strongly with CPGs and EBM use

Two multiple regression analyses were subsequently undertaken with domains set as independent variables and current and future use of CPGs set as dependent variables. Normal regression assumptions were ascertained.

Regression analyses' findings are summarised in Tables 4 and 5. These suggest that “confidence”, “familiarity” and “commitment and duty” had the strongest and most consistent associations with both current and future use of CPGs in decreasing strength order. “Time” correlated significantly with future use only.

**Table 4:** Regression of current CPG use variable against the five domains

	B	SE B	β
Constant	−1.305	.385	
“Commitment and duty”	.070	.014	.149*
“Time”	.023	.014	.048
“Familiarity”	.067	.016	.121*
“Confidence”	.097	.013	.219*
“Perceived benefits”	-.002	.019	-.003

*Note*: Adjusted R^2^ = .147

* *p* < .001

**Table 5:** Regression of intended future CPG use variable (three point collapsed version) against the five domains

	B	SE B	β
Constant	.103	.137	
“Commitment and duty”	.012	.005	.073***
“Time”	.014	.005	.081**
“Familiarity”	.041	.006	.206*
“Confidence”	.037	.005	.235*
“Perceived benefits”	-.020	.007	-.080**

*Note*: Adjusted R^2^ = .182

* *p* < .001, ** *p* < .01, *** *p* < .05

No association was noted between the “perceived benefits” domain and current use however a small but significant negative correlation was noted with future intended use.

## Discussion

### Principal findings

Firstly, we identified a generally high level of regard for EBP among Foundation Doctors in England and Wales. Both internationally and nationally, this is in keeping with existing research that notes favourable attitudes towards EBP irrespective of specialty and seniority [25]. This is in line with the UK Foundation Programme’s Curriculum on maintaining good medical practice [24].

A majority of doctors reported that CPGs reduced litigation and patient choice. This mirrors findings from our national medical student survey and may similarly reflect deficits in their knowledge of CPG development. A significant minority of doctors report low confidence in critically appraising evidence. This is in spite of the Foundation Programme Curriculum specifying competencies in CPGs and EBM that must be achieved in order to satisfactorily complete the 2-year programme [24].

Interestingly, we identified that respondents reported fear or guilt if CPG use was in conflict with senior advice or when patient care suffered as a result. This stands in contrast to a TDF - based interview study of Foundation Doctors investigating prescribing errors and existing literature on fear of medical litigation due to non-adherence to CPGs [26, 27]. Whilst a systematic review of GP attitudes towards CPGs did identify a fear of misdiagnosis, it was deemed to be unrelated to CPG use (i.e. all the participating physicians indicated that it did not matter whether they followed a CPG as long as they did not miss a diagnosis) [28].

Finally, we identified five behavioural domains (“confidence”, “familiarity”, “commitment and duty”, “time” and “perceived benefits”) of which “confidence”, “familiarity” and “commitment and duty” correlated with both current and future intention to use CPGs and “time” correlating with future intentions. Over the past decade, the field of implementation science had sought to explore the influence of other behavioural predictors beyond conventional barriers such as time in CPG and EBM use [29]. Amongst other frameworks, this led to the development and subsequent validation of the TDF [29]. This was therefore a surprising finding.

However, this was in keeping with a systematic review published in 1999 involving 76 articles that included 120 different surveys investigating 293 potential barriers to physician CPG adherence [30]. Here they identified poor familiarity, time and confidence as key barriers. Our study findings have therefore confirmed both the importance and persistence of traditionally researched barriers in improving uptake of CPGs.

We found that the TDF model was not useful in capturing the behavioural domains that influence implementation among our survey respondents. This was unexpected and stands in contrast to several other cross-sectional and qualitative studies that have successfully used the TDF to assess implementation difficulties [13–16]. However, this was in keeping with a cross-sectional study of dental healthcare providers which noted similar difficulties in obtaining satisfactory Cronbach’s alpha scores which required exploratory factor analyses to be performed [31].

### Strengths

To date, this is the largest study to quantify the relative importance of various factors in implementing EBM and CPGs in frontline clinical practice by junior doctors. This follows a qualitative interview study of 22 Foundation Doctors that identified seven behavioural domains from the TDF that could potentially be targeted to reduce prescribing errors [26]. Whilst we were unable to map our survey respondents to the TDF, by utilising the TDF to expand and develop our questionnaire, a wide breadth of factors influencing CPGs beyond traditionally researched barriers such as “knowledge” and “time” were explored [29, 32].

### Limitations

Despite an aggressive recruitment strategy of contacting (1) all Workforce Deaneries (*n* = 14), (2) hospitals (*n* = 167) in England & Wales and (3) recruiting Foundation Doctors as local champions, the response rate was poor at 12.9 % (*n* = 1693). This is in keeping with existing research that notes doctors as a group from which it is often difficult to obtain high response rates [33].

As a result of not having a high response rate, there is a possibility of our study receiving responses from a biased sample of doctors. This limits the generalisability of our findings to the national cohort of Foundation Doctors. In addition, we were unable to assess for social desirability bias amongst our survey respondents (i.e. respondents overstating their actual use of CPGs).

Furthermore, owing to the cross-sectional study design, it remains unknown whether their behavioural structure is stable over time. For example, research has noted less favourable attitudes towards CPGs with increasing both seniority of clinical practice and in different specialties [34, 35].

Nevertheless, due to the large sample size, they were sufficient to produce relatively accurate whole population estimates. For example, for the questions on proportion of respondents who use CPGs, the achieved sample size allows a calculation of a 95 % confidence interval (CI) of plus or minus 2.2 %. Future studies should consider the use of unconditional incentives to boost response rates [36].

### Implications for further research

Whilst the TDF was developed to encompass a broad range of different theories to arrive at a comprehensive framework that incorporates the main theoretical explanations for behaviour, it is however not an exhaustive list [12]. Unlike other studies that have successfully utilised the TDF to study the implementation of a defined guideline in a distinct healthcare professional group, we surveyed a large number of doctors from all specialties, each of which has a different approach towards both the development and use of CPGs.

One can therefore postulate that the TDF should not be utilised to investigate collective attitudes through a questionnaire study using closed-questions when a large response spectrum is anticipated. For example, the TDF was successfully used in a semi-structured interview study of 22 Foundation Doctors through use of an open-ended TDF-based topic guide that allowed interviewers to prompt participants to discuss their beliefs using general behavioural descriptions [26].

Another possible explanation is that while the questions included in the questionnaire were deliberately designed to “evoke” attitudes and feelings on the TDF domains among the participants, they were also inadvertently worded in such a way to also stimulate and tap into alternative attitudinal structures more strongly than anticipated.

From our experience, caution is advised when applying the TDF to a specific healthcare professional group without first attempting to validate the model. Use of a recently published generic questionnaire that can be tailored to suit different targets, actions, contexts, and times of interest with discriminant content validity established is recommended for future research [23].

### Implications for practice

In this large survey of newly qualified doctors, “confidence”, “familiarity” and “commitment and duty” were identified as domains that influence use of CPGs in frontline practice. In addition, a significant minority were not confident in critically appraising evidence.

The implications of our research are potentially two-fold, given the possibility that they could inform both undergraduate and postgraduate medical education in the UK. At the level of medical education, our identified behavioural domains could be used as a framework to inform targeting interventions to improve the uptake of CPGs [29].

Despite publication of the GMC’s Tomorrow’s Doctors which mandates training in evidence - based practice and a national NICE education strategy, our findings are in keeping with a 2009 survey of Foundation Doctors' preparedness for clinical practice that reported only 63.4 % being able to critically appraise relevant evidence [11, 37]. There therefore remains a need to provide support and training to those attempting to undertake EBM teaching.

Clinically integrated teaching has been reported to improve knowledge, skills, attitudes toward CPG and EBM [32]. Training could be nuanced with a focus on encouraging trainee doctors to have greater confidence about seamless guideline use in routine practice, to be more familiar with the concepts and the development of CPGs and to assert their interest in EBP.

## Conclusion

This exploratory survey of a large group of newly qualified doctors offers a hypothesis for what influences their implementation of CPGs and EBM. Whilst validated in several healthcare professional groups, the validity of the TDF should not be assumed for all groups.

In comparison with previous research, positive views of CPGs were generally noted amongst these doctors. Guideline developers, educators and implementers should be encouraged by these findings. Further scope to improve implementation remains possible however with a focus on addressing the identified factors as a way forward.

## Abbreviations

CPG: clinical practice guidelines
EBM: evidence - based medicine
GMC: General Medical Council
NHS: National Health Service
NICE: National Institute for Health and Care Excellence
