# Erratum to: What drives junior doctors to use clinical practice guidelines? A national cross-sectional survey of foundation doctors in England & Wales

**DOI:** 10.1186/s12909-016-0557-9

**Published:** 2016-02-05

**Authors:** Logan Manikam, Andrew Hoy, Hannah Fosker, Martin Ho Yin Wong, Jay Banerjee, Monica Lakhanpaul, Alec Knight, Peter Littlejohns

**Affiliations:** Population, Policy and Practice, UCL Institute of Child Health, 30 Guildford Street, London , WC1N 1EH,, UK; National Institute for Health and Care Excellence, 10 Spring Gardens, London , SW1A 2BU,, UK; Leicestershire Partnership NHS Trust, Bradgate Mental Health Unit, Glenfield Hospital, Groby Road, Leicester , LE3 9EJ,, UK; University College London Hospitals NHS Foundation Trust, 235 Euston Road, London , NW1 2BU,, UK; University Hospitals of Leicester NHS Trust, Infirmary Square, Leicester , LE1 5WW,, UK; King’s Improvement Science, Health Service and Population Research Department, Institute of Psychiatry, Psychology & Neuroscience, King’s College London, IoPPN Main Building, London , SE5 8AF,, UK; Department of Primary Care and Public Health Sciences, King’s College London, Addison House, London , SE1 IUL,, UK

## Erratum

During production of the original article [1], a document supplied by the author containing a set of further corrections to the manuscript was overlooked. The article therefore published containing a number of errors which are detailed here.

The publisher would like to apologise for these errors, which have all been corrected in the original article.

In the ‘Results’ section of the abstract, the number of responses received to the questionnaire was incorrectly given as 1698. This should have been 1693, as was correctly reported throughout the article itself.

The first names of all authors except Martin Ho Yin Wong were mistakenly abbreviated to a first initial. The original article has been updated with the correct, full first names, as they appear in this erratum.

The affiliation of Hannah Fosker was incorrect, and has now been updated to: Leicestershire Partnership NHS Trust, Bradgate Mental Health Unit, Glenfield Hospital, Groby Road, Leicester, LE3 9EJ, UK. In the original article, this has been inserted as affiliation 3, and subsequent affiliations have been renumbered to reflect this.

The affiliation of Alec Knight has also been amended to the correct address: King’s Improvement Science, Health Service and Population Research Department, Institute of Psychiatry, Psychology & Neuroscience, King’s College London, IoPPN Main Building, London SE5 8AF, UK.

The affiliation of Monica Lakhanpaul has been changed from affiliation 7 (Department of Primary Care and Public Health Sciences, King’s College London) to affiliation 1 (Population, Policy and Practice, UCL Institute of Child Health).

In the Acknowledgements section, it was stated that: “AK was supported by the National Institute for Health Research (NIHR) Collaboration for Leadership in Applied Health Research and Care South London at King’s College Hospital NHS Foundation Trust.”

This sentence should have included Peter Littlejohns, to read: “PL and AK were supported by the National Institute for Health Research (NIHR) Collaboration for Leadership in Applied Health Research and Care South London at King’s College Hospital NHS Foundation Trust.”

Finally, there were minor formatting errors throughout the article, mainly involving the inconsistent use of hyphens, which have now been corrected.

## References

[1] Manikam L, Hoy A, Fosker H, Wong MHY, Banerjee J, Lakhanpaul M (2015). What drives junior doctors to use clinical practice guidelines? A national cross-sectional survey of foundation doctors in England & Wales. BMC Med Educ.

